# Role of CD93 in Health and Disease

**DOI:** 10.3390/cells12131778

**Published:** 2023-07-04

**Authors:** Giovanni Tossetta, Federica Piani, Claudio Borghi, Daniela Marzioni

**Affiliations:** 1 Department of Experimental and Clinical Medicine, Università Politecnica delle Marche, 60126 Ancona, Italy; d.marzioni@univpm.it; 2 Cardiovascular Medicine Unit, Heart, Chest and Vascular Department, IRCCS Azienda Ospedaliero-Universitaria di Bologna, 40138 Bologna, Italy; federica.piani2@unibo.it (F.P.); claudio.borghi@unibo.it (C.B.); 3 Hypertension and Cardiovascular Risk Research Center, Medical and Surgical Sciences Department, Alma Mater Studiorum University of Bologna, 40138 Bologna, Italy

**Keywords:** CD93, angiogenesis, inflammation, AMD, polymorphism, SNP, cancer, C1q, C1qR1, C1qRp

## Abstract

CD93 (also known as complement protein 1 q subcomponent receptor C1qR1 or C1qRp), is a transmembrane glycoprotein encoded by a gene located on 20p11.21 and composed of 652 amino acids. CD93 can be present in two forms: soluble (sCD93) and membrane-bound (CD93). CD93 is mainly expressed on endothelial cells, where it plays a key role in promoting angiogenesis both in physiology and disease, such as age-related macular degeneration and tumor angiogenesis. In fact, CD93 is highly expressed in tumor-associated vessels and its presence correlates with a poor prognosis, poor immunotherapy response, immune cell infiltration and high tumor, node and metastasis (TNM) stage in many cancer types. CD93 is also expressed in hematopoietic stem cells, cytotrophoblast cells, platelets and many immune cells, i.e., monocytes, neutrophils, B cells and natural killer (NK) cells. Accordingly, CD93 is involved in modulating important inflammatory-associated diseases including systemic sclerosis and neuroinflammation. Finally, CD93 plays a role in cardiovascular disease development and progression. In this article, we reviewed the current literature regarding the role of CD93 in modulating angiogenesis, inflammation and tumor growth in order to understand where this glycoprotein could be a potential therapeutic target and could modify the outcome of the abovementioned pathologies.

## 1. Introduction

**Figure 1:**
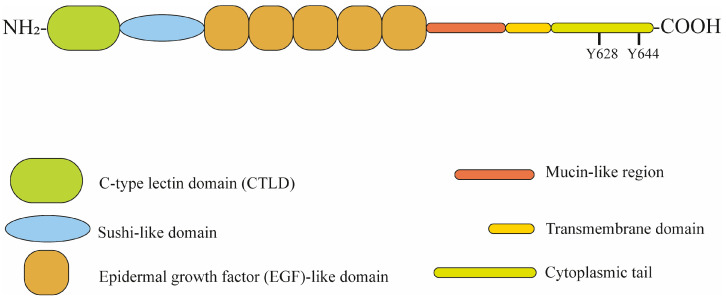
Localization of CD93 domains. Y = tyrosine.

Human CD93, also known as complement protein 1 q subcomponent receptor (C1qR1 or C1qRp), was believed to be the cell surface receptor for the complement component C1q, but it has been reported that CD93 was not able to engage C1q [1]. CD93 belongs to the C-type lectin domain (CTLD) group 14 family of transmembrane glycoproteins together with thrombomodulin, CLEC14A and CD248. This protein family has a similar ectodomain architecture and is involved in several cell processes including angiogenesis, inflammation and cell adhesion [2]. While thrombomodulin, CD93 and CLEC14A are mainly expressed by endothelial cells, CD248 is expressed by vasculature-associated pericytes, activated stromal fibroblasts, mesenchymal stem cells and tumor cells, but not in the endothelium. Since this protein family has been linked to tumor development (especially CD93, CLEC14A and CD248), there is a great interest in studying these proteins as possible therapeutic targets in cancer treatment [2,3]. CD93 is a transmembrane glycoprotein encoded by a gene located on 20p11.21 and composed of 652 amino acids organized in a N-terminal signal peptide, a C-type lectin domain (CTLD) containing eight conserved cysteine residues, a sushi-like domain, five epidermal growth factor (EGF)-like domains, a potential N-linked glycosylation site, a mucin-like region containing several predicted O-linked glycosylation sites, a transmembrane domain and a cytoplasmic tail containing a potential tyrosine kinase phosphorylation site [2]. CD93 structure organization is shown in Figure 1.

CD93 glycosylation has a key role in CD93 stability. In fact, the inhibition of the glycosylation of CD93 by treating human histiocytic lymphoma U937 cell line with benzyl 2-acetamido-2-deoxy-alpha-D-galactopyranoside (BAG) or by expressing CD93 in the Chinese hamster ovary (CHO)-K1 cell line (which are defective in protein glycosylation) caused a decrease in CD93 expression on the cell surface and the concomitant detection of CD93 in the culture media, demonstrating that the absence of glycosylation causes a rapid release of CD93 into the culture supernatant, proving a key role of CD93 O-glycosylation in the stable cell surface expression of CD93 [4]. Although CD93 is mainly expressed on endothelial cells, it has also been found expressed in neurons, hematopoietic stem cells, cytotrophoblast cells, monocytes, neutrophils, B cells, natural killer (NK) cells and platelets, suggesting an important role of CD93 also in these cells [5,6,7,8]. CD93 is also expressed in B cells (where it maintains antibody secretion [9]) and in tumor-associated vessels, where it is correlated with a poorer survival [10,11,12,13]. In fact, blocking angiogenesis in tumor-associated vessels can significantly reduce tumor growth, improving outcomes in patients with cancer [14].

This hypothesis is also validated by the fact that the silencing of CD93 by RNA interference in human endothelial cells significantly altered proliferation, migration, adhesion, sprout and tube formation of these cells [12,13,15]. This inhibitory effect on angiogenesis was also demonstrated by blocking CD93 with a monoclonal antibody against human CD93, which is a CD93 region between the CTLD and sushi domains [14]. It has also been demonstrated that CD93 could be significantly downregulated using brivanib alaninate, an inhibitor of vascular endothelial growth factor 2 (VEGFR2) and fibroblast growth factor 1 (FGF1) [16].

Although several extracellular matrix proteins such as collagen I and IV, gelatine, laminin, vitronectin and fibronectin have been proposed as potential ligands of CD93 [17], only multimerin 2 (MMRN2) has been found to interact with CD93 [18]. Moreover, it has been demonstrated that this interaction of MMRN2 with CD93 is dependent on the CTLD domain of CD93 [19]. This study also explains the antiangiogenic results obtained with the monoclonal antibody previously described, since this antibody binds a part of CTLD domain, interrupting CD93–MMRN2 interaction. In addition to the membrane-associated form, CD93 can also be present as soluble form (sCD93) which is detectable in blood. This soluble form of CD93 showed important functions, acting as a proangiogenic factor [20], opsonin [21] and diagnostic/prognostic factor in metabolic and inflammatory diseases [22,23].

The aim of this review is to provide an overview of the current literature regarding the multifaceted role of CD93 in efferocytosis and angiogenesis, but also in pathological conditions such as tumors, age-related macular degeneration (AMD) and inflammatory diseases.

## 2. CD93 in Physiological Conditions

### 2.1. Efferocytosis

Efferocytosis is an essential process in tissue homeostasis since it can prevent inflammatory and autoimmune diseases caused by apoptotic cell lysis. In fact, efferocytosis favors the phagocytic removal of apoptotic cells avoiding inflammation [21]. CD93 plays an important role in efferocytosis since mutations in CD93 predispose patients to efferocytosis-associated diseases such as coronary heart disease, which is caused by the accumulation of apoptotic cells beneath the cardiac vasculature [21,24]. Norsworthy and colleagues found that CD93 was expressed at very low levels in resident peritoneal macrophages and its expression depended on the activation state of the macrophages. Moreover, by using CD93-deficient mice (CD93^−/−^), the authors proved that the phagocytic ability of the macrophages from CD93^−/−^ mice was unaffected by the absence of CD93, suggesting that the lack of CD93 had no effect on these C1q-mediated events. However, CD93^−/−^ mice showed phagocytic defects in the clearance of apoptotic cells, although no variation was found in the engulfment ability of apoptotic cells in CD93^−/−^-derived mouse macrophages. Thus, although CD93 is expressed on the peritoneal macrophage, it is not involved in C1q-mediated enhancement of phagocytosis, but it may contribute to the in vivo clearance of dying cells [25]. Another study used the THP-1 cell line and human monocytes and macrophages to demonstrate that sCD93 potently opsonizes apoptotic cells, whereas membrane-bound CD93 showed no phagocytic or efferocytic activity. Moreover, the authors found that integrin subunit αxβ2 acts as the receptor for sCD93, and sCD93 binds to apoptotic cells via its C-type lectin-like domain and to αxβ2 by its EGF-like repeats. Thus, sCD93 acts as an opsonin that binds apoptotic cells and phagocytic integrin subunit αxβ2 on macrophages, enhancing macrophages’ efferocytosis [21].

Thus, CD93 does not interact with C1q and does not alter C1q-mediated phagocytosis. However, CD93 is expressed at very low levels in tissue macrophages and its expression depends on the activation status of these cells. Furthermore, although CD93 does not affect the phagocytic activity of macrophages, it may contribute to the in vivo clearance of dying cells.

### 2.2. Angiogenesis

The expression on the endothelial cell surface of molecules involved in cell adhesion plays a key role during angiogenesis since they can favor the growth and survival of new vessels [26,27,28]. During the spreading of endothelial cells, the Src kinase phosphorylates the cytoplasmic tail of CD93 on tyrosine 628 and 644 favoring the binding of the adaptor protein Cbl, which regulates cell migration [17]. In migrating cells, the phosphorylation of Cbl on tyrosine 774 causes the activation of Rho proteins favoring cytoskeleton remodeling [29]. Galvagni and colleagues investigated the mechanism of action of CD93 in promoting angiogenesis using proteomic analyses. The authors found that silencing CD93 in endothelial cells caused an increase in dystroglycan expression, a laminin-binding protein involved in angiogenesis and overexpressed in vascular endothelial cells within malignant tumors. Interestingly, the authors found that the interaction between β-dystroglycan and CD93 could promote endothelial cell migration and organization into capillary-like structures. Moreover, it was demonstrated that the phosphorylation of CD93 on tyrosine 628 and 644 after cell adhesion on laminin through dystroglycan was necessary for the acquisition of the endothelial migratory phenotype. Additionally, during cell spreading the phosphorylated CD93 could recruit the protein Cbl, implicated in cell adhesion and organization of the actin cytoskeleton, which in turn was phosphorylated, inducing cell migration, demonstrating a key role of CD93 in modulating endothelial cell migration [17].

The role of CD93 in regulating cytoskeletal dynamics during angiogenesis was also investigated by Barbera and colleagues. In fact, they found that the phosphorylation of CD93 was Src-dependent and that the adaptor protein Cbl induces the recruitment of Crk, an activator of the Rho/Rac family of small GTPases involved in the regulation of intracellular actin dynamics [30]. In fact, the authors found that CD93 coordinates the activation of RhoA and Rac1 at the cell edge of spreading cells favoring cell motility [31]. Thus, CD93 is an important endothelial cell adhesion molecule involved in regulating vascular maturation. In the proliferating endothelium, the interaction between CD93 and the extracellular matrix induces the activation of many signaling pathways involved in cell adhesion, migration and vascular maturation.

It is known that during cell spreading, cells undergo an apical-basolateral polarization, causing a strong rearrangement of the cytoskeleton [32]. An interesting study identified the recycling pathway of CD93 following endothelial cells’ adhesion and migration. The authors found that, at the onset of endothelial cell spreading, CD93 is localized in the apical bud, a membrane moesin-rich domain able to mediate polarized trafficking through the branching out of linear actin cables [33]. Moreover, the interaction of the cytoplasmic domain of CD93 with moesin and F-actin is essential for CD93 retrieval in adhering and migrating cells. Further, the small GTPase Rab5c is a key component of the molecular machinery involved in CD93 recycling to the endothelial cell surface. In fact, in the Rab5c endosomal compartment, CD93 forms a complex with Multimerin-2 and active β1 integrin, which is recycled back to the basolaterally polarized cell surface via clathrin-independent endocytosis [34]. This endocytic pathway of CD93 sustains previous studies proving that integrins and their ligands have a high traffic-dependent turnover and Rab5 can regulate β1 integrin internalization. This process is very efficient because it allows cells to respond early and rapidly to stimuli coming from the extracellular environment [35,36]. Vascular permeability to plasma is an essential mechanism to maintain tissue homeostasis and endothelial cell-to-cell junctions play a key role in regulating molecular trafficking between blood and tissue [37]. It has been reported that CD93 interacts with vascular endothelial (VE)-cadherin, limiting its phosphorylation and turnover. In fact, CD93 deficiency in endothelial cells and CD93^−/−^ mice induced VE-cadherin phosphorylation, allowing its internalization and increasing the blood–brain barrier permeability. Interestingly, the authors proved that these effects of CD93 in regulating endothelial barrier properties were due to the activation of Rho/Rho kinases [38]. An interesting study showed that treatment of endothelial cells with recombinant CD93 protein promoted tube formation and sprouting. Moreover, recombinant CD93 accelerated wound healing, promoting the formation of vascular-like structures in mouse subcutaneous tissue. Conversely, CD93 deficiency in CD93^−/−^ mice delayed wound repair due to a reduced neovascularization of the wound area [39]. Another study evaluated the possible angiogenic effects of soluble CD93 generating two recombinant-soluble CD93 proteins with EGF-like domains: rCD93D123 (containing domains 1, 2 and 3) and rCD93D23 (containing domains 2 and 3). The authors found that rCD93D23 was more efficient than rCD93D123 at stimulating the proliferation and migration of human endothelial cells. Moreover, the authors found that the production of matrix-metalloproteinase 2 (MMP2) significantly increased after treating endothelial cells with rCD93D23. Further, rCD93D23 treatment induced cell differentiation of endothelial cells activating focal adhesion kinase (FAK), phosphoinositide 3-kinase (PI3K)/Akt/endothelial nitric oxide synthase (eNOS) and extracellular signal-regulated kinases-1/2 (ERK1/2) signaling (three important signaling pathways responsible for cell migration, proliferation, permeability and homeostasis of endothelial cells [40]) in the endothelium, promoting blood vessel formation in vivo and in vitro. Thus, soluble CD93 is a potent angiogenic factor [20].

MicroRNAs (miRNAs) are short (about 20 nucleotides) endogenous noncoding RNAs involved in the post-transcriptional regulation of gene expression. miRNAs play a key role in regulating various biological processes and are involved in the onset and progression of several cancerous [41,42,43,44] and non-cancerous diseases [45,46,47,48]. Therefore, miRNA inhibition could be a promising therapeutic treatment for many diseases. MRG-110 is an antisense oligonucleotide targeting miR-92a-3p, an miRNA involved in vascular homeostasis [49,50]. Interestingly, it has been reported that an intravenous administration of MRG-110 significantly decreased miR-92a-3p levels in whole blood and peripheral blood mononuclear cell (PBMC)-derived CD31^+^ cells (which comprise monocytes and circulating endothelial cells) of healthy subjects. Importantly, MRG-110 treatment significantly increased the expression level of CD93 in whole blood, suggesting that MRG-110 could inhibit miR-92a in the peripheral blood, derepressing CD93 expression. However, the authors did not evaluate CD93 expression in PBMC-derived CD31^+^ cells. Thus, CD93 could be a target of miR-92a [51]. This hypothesis has been indirectly verified by Gallant-Behm and colleagues. In fact, they transfected endothelial cells with MRG-110 and found a significant increase in CD93 expression, proving that MRG-110 can efficiently modulate CD93 expression [52].

Looking at the data discussed in this section (summarized in Table 1), we can conclude that CD93 plays a key role in regulating angiogenesis through its interactions with β-dystroglycan, VE-cadherin and MMRN2 that facilitate endothelial cell adhesion and migration. Moreover, CD93 expression can be regulated by non-coding RNA, such as miR-92a-3p and MRG-110. It has been also demonstrated that sCD93 has important angiogenic properties.

## 3. CD93 in Diseases

### 3.1. Role of CD93 Gene’s Single-Nucleotide Polymorphisms (SNPs)

The term single-nucleotide polymorphism (SNP) refers to the presence in two or more variant forms of a specific DNA sequence in different individuals. SNPs can be found within the coding regions of genes altering the amino acid sequence of the encoded protein and impairing its function [53,54]. SNPs can also be located in the 5′ or 3′ untranslated regions (UTR) of genes, altering the gene expression by affecting regulatory elements or mRNA stability [53,54,55].

*CD93* gene can be interested by three single-nucleotide polymorphisms (SNPs): rs2749817, rs2749812 and rs3746731 (see Table 2). The SNP rs2749817 is located downstream of the *CD93* gene’s 3′ untranslated region (UTR), while rs2749812 is located at *CD93* gene’s 3′ UTR, and rs3746731 is located in exon 1 in the *CD93* gene. Both these SNPs can potentially influence the *CD93* gene’s translation and posttranslational modifications [10,24,56].

Psoriasis is an autoimmune disease characterized by inflammation and increased angiogenesis [57,58]. Shehata and colleagues investigated the role of CD93 in modulating inflammation in this pathology and found that CD93 expression was increased in both endothelial and inflammatory cells of patients affected by psoriasis. Moreover, T/C and C/C genotypes of the SNP rs2749817 of *CD93* genes were lower in patients compared with controls. Interestingly, the C/C genotype was present only in some cases [59]. Another study found that the expression of the *CD93* gene was increased in lesioned and non-lesioned skin of patients with psoriasis. Moreover, immunohistochemical analysis showed that CD93 was expressed in dermal endothelial cells in lesioned skin. Furthermore, psoriasis was associated with rs2749817 but not with the rs2749812 polymorphism. No significant differences in CD93 protein levels were found in serum or in PBMCs between psoriasis patients and controls [60]. Thus, the increased CD93 expression of psoriatic skin may be associated with the rs2749817 polymorphism, suggesting an important role of CD93 in psoriasis disease pathogenesis. Interestingly, another study found that rs2749812 was associated with increased plasma sCD93 levels and increased CD93 mRNA expression in coronary artery disease [56,61]. Furthermore, the T/T genotype of rs2749817 was commonly found in patients with stage IV colorectal cancer (CRC), which is characterized by a higher risk of death and recurrence [10]. Another study evaluated the role of SNP rs3746731 of the *CD93* gene in patients with familial hypercholesterolemia and studied the association between this SNP and coronary heart disease (CHD). This study reported an association between the presence of rs374673 and CHD onset. Thus, the presence of this SNP could predict CHD in patients with familial hypercholesterolemia [24].

The studies discussed in this section showed that the C/C genotype of the SNP rs2749817 of CD93 was associated with psoriasis onset, while the T/T genotype of rs2749817 was commonly found in patients with stage IV CRC. Moreover, both rs374673 and rs2749812 have been associated with coronary artery disease.

### 3.2. Age-Related Macular Degeneration (AMD)

Age-related macular degeneration (AMD) is among the major causes of vision loss, especially among the elderly [62,63]. Depending on the progression and symptoms, AMD is classified in early, intermediate and advanced disease [64]. The latter form of AMD is due to the advanced neovascularization of choriocapillaris, the blood vessels located posterior to the outer retina, which leads to vascular leakage, hemorrhage and fibrosis [65]. For this reason, blocking neovascularization with intraocular injections of anti-Vascular Endothelial Growth Factor (VEGF) drugs is currently the main treatment for this form of AMD [66]. Although these treatments significantly improve AMD progression, they require frequent injections because the treatment does not lead to a persistent regression of the neovascularization [66]. Thus, a more efficient therapy is needed.

An interesting study showed that the expression of CD93 and MMRN2 is significantly increased in the hyperproliferative choroidal endothelial cells of AMD patients compared with that of healthy controls. Moreover, neovascularization was significantly reduced in CD93^−/−^ mice exposed to laser photocoagulation. Interestingly, the use of antibodies that hamper the CD93/MMRN2 interaction significantly reduced vascular sprouting in the human choroidal endothelial cells, demonstrating that the interaction of CD93 with Multimerin-2 plays a key role in pathological vascularization of the choroid, suggesting new therapeutic approaches in the treatment of AMD [67].

Another interesting study confirmed that choroidal neovascular membranes from AMD patients displayed a strong CD93 staining. Similar results were found in intraocular and extraocular humor vessels, whereas blood vessels in normal choroids displayed only a weak CD93 staining. Interestingly, the authors found that sCD93 levels were increased in the aqueous humor of AMD patients and tended to decrease (not statistically significantly) after treatment with anti-VEGF drugs. Thus, the increased expression of the transmembrane and sCD93 in patients with neovascular AMD suggests CD93 as a potential antiangiogenic target in AMD treatment [68].

Looking at the studies discussed in this section, we can conclude that CD93 plays a key role in promoting the neovascularization of choriocapillaris in patients with AMD and that this effect can be blocked by using antibodies that hamper the CD93/MMRN2 interaction. This is an important finding, since the combination of antibodies targeting CD93/MMRN2 interaction and VEGF may significantly improve the treatment of AMD patients.

### 3.3. Tumors

Tumor angiogenesis plays a key role in solid cancers’ growth [69,70,71]. Therefore, characterizing the mechanisms regulating vascular stability is of upmost importance to develop specific drugs that can improve outcomes for these patients.

Cancer immunotherapy is an efficient therapy with minimal side effects used to treat many types of cancer. This therapy is based on the use of compounds that can activate or boost the immune response to kill cancer cells. These compounds include immune checkpoint inhibitors (ICIs) (e.g., PD1/PD-L1), cytokines (e.g., IL-2, TGF-β1, IL-12 and IL-15), chimeric antigen receptor (CAR)-T cells and monoclonal antibodies [72]. Although immunotherapy significantly improves tumor progression and patient survival, several patients do not show any beneficial effects from this therapy [73].

It has been reported that CD93 is also associated with the infiltration of immune cells in tumor tissue and immunotherapy responses in cancer patients [74]. Interestingly, Tong and colleagues found a significant increase in CD93 expression in tumor tissues compared with the adjacent normal tissues in several types of cancer. Moreover, a high CD93 expression was associated with tumor angiogenesis, immune cell infiltration, a poor prognosis and high TNM stage in many cancer types. However, a high CD93 expression showed a protective effect in kidney renal clear cell carcinoma (KIRC), an aggressive form of renal cell carcinoma [75]. Therefore, since CD93 can regulate immune responses and immune cell infiltration, it can significantly regulate the malignant properties of various cancer types and it could have a potential value as a biomarker for determining the prognosis and immune infiltration in various type of cancers [76].

Although the therapy with immune checkpoint inhibitors significantly improved cancer treatment, many patients do not show clinical benefit [77,78]. However, it has been shown that combining immunotherapy with CD93-blocking agents can sensitize tumors to immune-checkpoint blocker therapy, improving therapy response in preclinical tumor models [79]. Zhang and colleagues found that an increased expression of CD93 was associated with a poor prognosis in cancer patients and was correlated with mismatch repair (MMR) gene expression, tumor mutation burden (TMB), microsatellite instability (MSI) and immune cell infiltration. Functional analysis showed a correlation among CD93 and cancer promotion, angiogenesis and inflammation. Thus, CD93 could be a good prognostic marker and molecular target in many types of cancer [80].

An immature vasculature within solid tumors represents an important obstacle to immunotherapy since the resulting hypoxia limits immune cell infiltration [81,82,83,84]. Sun and colleagues found that insulin-like growth factor binding protein 7 (IGFBP7), an extracellular matrix (ECM) protein present on tumor vasculature, interacts with CD93, contributing to an abnormal tumor vasculature. In fact, the authors showed that IGFBP7 and CD93 expression was significantly increased in tumor-associated endothelial cells. Moreover, the use of monoclonal antibodies able to block CD93/IGFBP7 interaction in mouse tumor models promoted vascular maturation, reducing tumor hypoxia and increasing tumor perfusion. This effects significantly increased drug delivery in tumor tissue, improving antitumor response to gemcitabine and fluorouracil. Moreover, the blockade of the CD93 pathway increased intratumoral effector T cells, sensitizing mouse tumors to immune checkpoint therapy. Accordingly, overexpression of IGFBP7 and CD93 caused a poor response to the therapy with programmed cell death 1/ligand 1 (PD-1/PD-L1) checkpoint inhibitors. Thus, the blockade of IGFBP7/CD93 interaction can be a novel approach to favor cancer treatment [79].

Glioblastoma is an aggressive form of brain tumor characterized by an abnormal and hyperpermeable vasculature [85]. An interesting study found that CD93 protein and gene expression was increased in tumor-associated blood vessels of human glioma compared with that in a normal brain. Moreover, CD93 expression was correlated to the tumor grade with a maximum expression in grade IV glioma (glioblastoma) vessels, suggesting a key role of CD93 tumor vascularization [11]. Another study evaluated the role of CD93 in regulating glioblastoma angiogenesis. In this study, the authors found that vascular expression of CD93 was correlated with poor survival in patients with high-grade astrocytic glioma. Moreover, intracranial growth in the GL261 mouse model of glioma was reduced in CD93^−/−^ host mice, improving survival compared with wild-type mice. Interestingly, the authors found that this effect was associated with increased vascular permeability and decreased vascular perfusion of tumor cells caused by a decreased vessel functionality due to the absence of CD93. The silencing of CD93 in endothelial cells induced displacement and loss of VE-cadherin from the cell–cell junctions as well as decreased cell–matrix adhesion and cell spreading due to an increased stress fiber formation and cytoskeleton rearrangement. In addition, researchers have found a reduced VEGF-induced tube formation in endothelial cells when CD93 was silenced, demonstrating the key role of CD93 in glioma angiogenesis and vascular function [12]. Further, investigating CD93 function in glioma, Lugano and colleagues confirmed that CD93 expression was increased in tumor vessels of high-grade glioma. Moreover, they found that CD93 regulated β1 integrin during tumor vascularization. In addition, CD93 was expressed in endothelial filopodia and promoted filopodia formation in endothelial cells and mouse retina. The CD93 expression on endothelial filopodia was stabilized by interactions with MMRN2, and their interaction (CD93/MMRN2 complex) was required for the activation of β1 integrin and phosphorylation of focal adhesion kinase (FAK) in endothelial cells [86].

Bao and colleagues investigated the role of CD93 in regulating nasopharyngeal carcinoma (NPC) progression and found an increased CD93 expression in NPC. Moreover, CD93 expression was correlated with the T classification, N classification, distant metastasis, clinical stage and poor prognosis. The authors also found that silencing CD93 in endothelial cells significantly inhibited angiogenesis. Moreover, silencing CD93 in the NPC cell line CNE2 significantly reduced cell proliferation, demonstrating the key role of CD93 in NPC progression and angiogenesis, and validating this protein as a novel therapeutic target in NPC [13].

Looking at the data discussed in this section (summarized in Table 3), we can speculate that CD93 may have an immunosuppressive role in tumor environments since it can limit immune cell infiltration, favoring the immune-escape of tumor cells. In addition, since CD93 is highly expressed in vascular networks within solid tumors, it can favor tumor vascularization and growth. Moreover, blocking CD93 with specific agents (e.g., monoclonal antibodies, non-coding RNA, etc.) can favor immunotherapy by increasing vascular permeability and immune cell infiltration in tumor tissue.

### 3.4. Inflammatory Diseases

Inflammation is a complex and tightly regulated biological response of body tissues to dangerous stimuli such as pathogens, damaged cells and irritants that activates immune cells and several molecular mediators to eliminate the cause of cell injury and re-establish a normal tissue homeostasis [87,88]. Although inflammation is a key regulator of tissue homeostasis, excessive and prolonged inflammation is involved in several pathological conditions such as diabetes [89,90], musculoskeletal disorders [91], neurological diseases [92,93], cardiovascular diseases [94,95], pregnancy complications [89,96,97,98,99] and cancer progression [100,101,102]. Thus, understanding the mechanisms involved in regulating inflammation is necessary to better treat these pathologies.

An interesting study by Jeon and colleagues evaluated the effect of sCD93 on THP-1 monocytic cells and human primary monocytes. In particular, authors evaluated the role of the mucin domain of sCD93 by treating these cells with two recombinant forms of human sCD93: hsCD93-Fc (carrying the CRD and EGF domains of human CD93 fused with human IgG1 Fc) and hsCD93-Mucin-Fc (which also carries the mucin domain). The authors found that the mucin domain was dispensable for the CD93 activity since both the recombinant forms of sCD93 could induce cytokine production in these cells. Moreover, the authors reported that hsCD93-Fc (authors used this form for all other experiments) was able to bind to the cell surface of THP-1 cells and human primary monocytes, indicating that monocytes express a putative receptor for sCD93. Treatment with hsCD93-Fc also induced differentiation of monocytes to macrophage-like cells since sCD93 treatment could induce cell adhesion and increase phagocytic activities. This differentiation resulted in an enhanced response to the stimulation of Toll-Like Receptors (TLRs) by LPS, increasing the production of proinflammatory cytokines such as IL-6, IL-1β and TNF-α. Interestingly, the authors also found increased sCD93 levels in synovial fluid from patients with rheumatoid arthritis, a chronic inflammatory disease [91], compared with synovial fluid from patients with osteoarthritis, where inflammation plays a secondary role [91,103]. Thus, sCD93 plays a key role in stimulating monocytes during inflammatory processes such as those ones found in rheumatoid arthritis [104].

Another study investigated the stimuli involved in CD93 shedding from human monocytes and neutrophils and found that the ectodomain of CD93 expressed on human monocytes and neutrophils was sensible to phorbol Dibutyrate (PDBu)-induced shedding, giving rise to a soluble fragment containing the domains from the N-terminal to the EGF repeats. Interestingly, the authors found that CD93 shedding was reduced when metalloproteinases were inhibited with 1,10-phenanthroline (a metalloproteases inhibitor), but shedding was not due to the TNF-alpha-converting enzyme (TACE, also known as ADAM17). Moreover, CD93 shedding on monocytes was followed by a decreased expression of CD93 on the cell surface, while neutrophils showed an increase in CD93 surface expression, suggesting that CD93 shed from the neutrophil surface was rapidly replaced by CD93 from intracellular stores. In addition, TNF-α and LPS significantly stimulated ectodomain cleavage of CD93 from monocytes. Thus, CD93 shedding can be trigged by multiple stimuli [105].

Peritonitis is an important inflammatory process involving the peritoneum, the serous membrane that lines the abdominal cavity and numerous organs contained therein [106]. Greenlee-Wacker and colleagues investigated the role of CD93 in inflammation and found that CD93^−/−^ mice showed an increase in leukocyte infiltration during thioglycolate-induced peritonitis due to an impaired vascular integrity in these mice. However, the authors did not find any differences in cytokine or chemokine levels in these mice. C1q-hemolytic activity in CD93^−/−^ mice was significantly decreased after thioglycolate injection, suggesting an alteration in the classical complement pathway. Interestingly, leukocyte recruitment and C1q-hemolytic activity was restored when CD93 was expressed on either hematopoietic cells or nonhematopoietic cells in bone marrow chimeric mice (the bone marrow of CD93^−/−^ mice was replaced with that from wild-type mice). Moreover, increased sCD93 levels in inflammatory fluid were observed only when CD93 was expressed on nonhematopoietic cells. Since cell-associated CD93 could restore a normal inflammatory response, these data suggest that cell-associated CD93, and not sCD93, is involved in the regulation of leukocyte recruitment and complement activation during murine peritonitis [107].

The role of CD93 has also been Investigated in neuroinflammation, an inflammatory process involving the nervous system. Griffiths and colleagues knocked out CD93 in two mice models of neuroinflammation; the MOG-experimental autoimmune encephalomyelitis (EAE) model and the antibody-dependent EAE (ADEAE) model. In CD93-expressing mice, CD93 was expressed in neurons, endothelial cells and microglia, while astrocytes and oligodendrocytes did not express CD93. Knock out of CD93 in both EAE and ADEAE mice showed more intense brain and spinal cord inflammation. Damage to and leakage through the blood–brain barrier was increased in CD93-deficient mice and was associated with a stronger neuronal injury when compared with wild-type EAE mice. Thus, CD93 is an important neuro-immune regulator to control central nervous system inflammation [108].

It has been reported that a GAIP-interacting protein, C terminus (GIPC), can interact with the cytoplasmic tail of CD93 in human monocytes [109]. An interesting study showed that inducing neuroinflammation via lipopolysaccharide (LPS) injection into the lateral ventricle of rats’ brains led to an increase in CD93 expression on cell membranes in the cerebral cortex. Interestingly, after inflammation induction, CD93 expression increased and then reduced, with distinct staining in the cytoplasm and nucleus. Moreover, the authors showed a colocalization of CD93 and GIPC, proving an interaction between these proteins, although no changes in GIPC expression were found during inflammation. CD93 was mainly expressed in microglial and neuronal cell membranes, while GIPC was expressed in both the cell membrane and cytoplasm. Thus, since the expression of CD93 quickly increased after LPS treatment, CD93 may participate in the early stage of central nervous system inflammation [110].

Another important disease where inflammation plays a key role is systemic sclerosis (SSc), a disease characterized by autoimmunity, fibrosis of the skin and internal organs, vasculopathy and inflammation [111]. An interesting study reported that serum sCD93 levels were significantly increased in patients with SSc compared with those of healthy individuals. Moreover, patients with diffuse cutaneous SSc showed higher levels of sCD93 than those with limited cutaneous SSc or systemic lupus erythematosus, and serum sCD93 levels were positively correlated with the severity of skin sclerosis. Interestingly, the authors found that sCD93 levels significantly decreased in parallel with improvement in skin sclerosis. The authors also found increased CD93 immunostaining on endothelial cells in lesioned skin tissues. Thus, CD93 may significantly contribute to the development of skin fibrosis in SSc [23].

Protein kinase C (PKC) isoenzymes consist of a large family of serine/threonine protein kinases that can be categorized in three major subfamilies: the classical PKC isoenzymes (cPKCs) that are regulated by Ca2 and diacylglycerol (DAG), the novel PKC isoenzymes (nPKCs) that are responsive to DAG but Ca2-independent, and the atypical PKC isoenzymes (aPKCs) that are independent of both Ca2 and DAG for their activation. Furthermore, the cPKCs and nPKCs, but not the aPKCs, can respond to phorbol myristate acetate (PMA) exposure [112,113]. It is known that PKCs can modulate many signal transduction pathways that are involved in the regulation of immune system responses [114]. Ikewaki and colleagues evaluated the role of PKC in the modulation of CD93 expression on the cell surface of human monocyte-like cell line (U937), human NK-like cell line (KHYG-1) and endothelial cells by using four types of protein kinase inhibitors: the classical protein kinase C (cPKC) inhibitor Go6976, the novel PKC (nPKC) inhibitor Rottlerin, the protein kinase A (PKA) inhibitor H-89 and the protein tyrosine kinase (PTK) inhibitor herbimycin A. The authors found that the nPKC inhibitor Rottlerin strongly downregulated CD93 expression on the U937 cells, whereas little or no effect was found in the treatments with the other inhibitors. However, CD93 expression was downregulated by Go6976, but not by Rottlerin, in the KHYG-1 cells and by both Rottlerin and Go6976 in endothelial cells. The PKC stimulator PMA strongly increased CD93 expression on the cell surface of all three cell-lines and induced interleukin-8 (IL-8) production by the U937 cells and interferon γ (IFNγ) production by the KHYG-1 cells. In addition, both Go6976 and Rottlerin significantly inhibited the upregulation of CD93 expression induced by PMA and IL-8 or IFNγ production in the respective cell-lines. Recombinant tumor necrosis factor α (rTNF-α) slightly upregulated CD93 expression on the U937 cells; recombinant interleukin-1β (rIL-1β), recombinant interleukin-2 (rIL-2), recombinant IFNγ (rIFNγ) and lipopolysaccharide (LPS) had no effect. Thus, PKC isoenzymes are involved in the regulation of CD93 expression on these cells and this regulation may interest the potential tyrosine kinase phosphorylation site contained in the cytoplasmic tail of CD93 [115].

The studies discussed in this section (summarized in Table 4) showed that serum sCD93 levels were increased in patients with SSc. Moreover, sCD93 induced IL-6, IL-1β and TNF-α production and the differentiation of monocytes to macrophage-like cells. sCD93 levels were also higher in synovial fluid from patients with rheumatoid arthritis. In addition, TNF-α and LPS stimulated CD93 shedding from monocytes. Importantly, loss of CD93 expression in inflammatory diseases caused an increased leukocyte infiltration due to an impaired vascular structure. Thus, targeting CD93 in inflammatory diseases may significantly reduce inflammation.

### 3.5. Cardiovascular Disease (CVD)

Cardiovascular disease (CVD) is due to vascular endothelial cells’ dysfunction frequently caused by diabetes, hypertension and hyperlipidemia [116]. The role of CD93 in CVD has been described in a dedicated systematic review published by our research group [117]. In this paragraph we briefly describe the studies discussed in our previous paper.

Regarding the SNPs of CD93, it has been reported that the SNP rs2749812 of CD93 was associated with a significantly higher cardiovascular risk [61] and the presence of hypertension at high-altitudes [118], while the SNP rs3746731 of *CD93* gene was also associated with an increased risk of developing coronary heart disease after adjustment for hypertension, diabetes mellitus, BMI, plasma HDL cholesterol and plasma triglyceride levels [24].

In addition to the SNPs of the *CD93* gene, the circulating levels of sCD93 were also associated with CVD. In fact, it has been reported that circulating sCD93 levels were higher in patients who suffered from ischemic stroke compared with control subjects [119] and were significantly associated with an increased mortality at 90 days after suffering an ischemic stroke [120]. Moreover, circulating sCD93 levels were significantly higher in patients with acute myocardial infarction than in control subjects [121].

Contrasting results were reported regarding type 2 diabetes. In fact, a study reported that circulating sCD93 levels were significantly lower in subjects (901 patients) with type 2 diabetes and were positively correlated with adiponectin and vitamin D, but inversely with BMI, insulin and HOMA [122]. These results were in contrast with the study of Lee and colleagues, reporting that subjects with type 2 diabetes (97 patients) had higher sCD93 levels, a lower estimated glomerular filtration rate (eGFR), higher albumin-to-creatinine ratio (ACR) and a higher risk of developing diabetic nephropathy [22]. These differences regarding the sCD93 levels in these studies could be due to the difference in sample size.

Regarding heart failure, an interesting study reported that patients with left heart failure and pulmonary hypertension had higher levels of sCD93 compared with healthy controls. Moreover, sCD93 significantly decreased after heart transplantation, reaching the levels of healthy controls [123]. These results are in agreement with the study by Bouwens and colleagues reporting that sCD93 levels were higher both at baseline and at follow-up of patients with heart failure [124].

Thus, evaluation of CD93 SNPs presence and/or sCD93 levels in patients at risk of CVD may significantly improve or avoid CVD onset, allowing an early treatment (when possible) of these patients.

## 4. Conclusions

**Figure 2:**
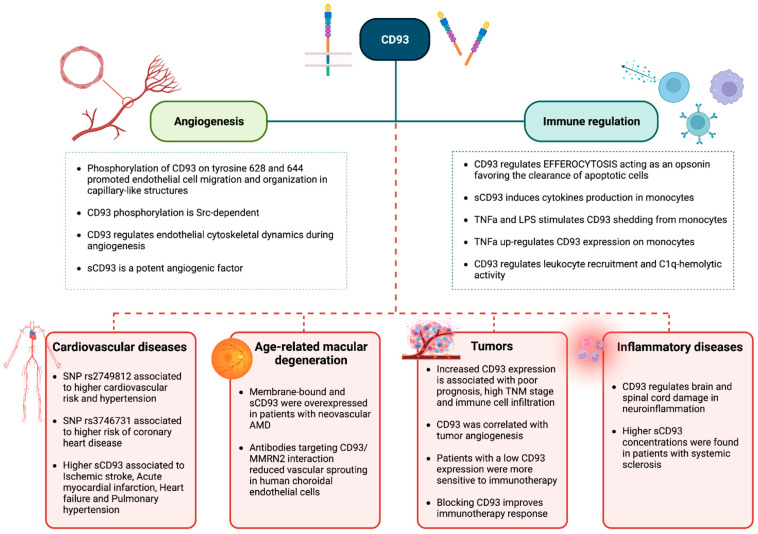
Summary of CD93 functions in health and diseases.

In this review we showed that CD93 plays important cell functions in physiological and pathological conditions (summarized in Figure 2). Moreover, we showed that the presence of SNPs may play an important role in regulating CD93 expression. In fact, we showed that SNP rs2749817 was highly associated with psoriasis [59] and colorectal cancer [10] onset, while rs2749812 was associated with coronary artery disease [56,61]. Moreover, the presence of SNP rs3746731 in patients with familial hypercholesterolemia was associated with coronary heart disease (CHD) onset in these patients [24]. We also showed an involvement of CD93 in age-related macular degeneration (AMD), with it being highly expressed in the choroidal endothelial cells of these patients. Thus, CD93 may be a potential therapeutic target since antibodies or compounds blocking CD93 expression could inhibit neovascularization in these patients. CD93 is also involved in regulating tumor vascularization. In fact, CD93 expression was significantly higher in tumor-associated vessels. Moreover, CD93 expression was associated with poor prognosis, immune cell infiltration and high TNM stage in many cancer types. Moreover, low CD93 expression showed an improved cancer outcome and immunotherapy response (see Table 3).

CD93 was also involved in important inflammatory-associated diseases such as systemic sclerosis and neuroinflammation. In fact, both CD93 forms (soluble and membrane-bound) were significantly higher in inflammatory diseases and sCD93 was able to activate monocytes, favoring the production of inflammatory cytokines. Moreover, inflammation could induce CD93 shedding (see Table 4).

**Figure 3:**
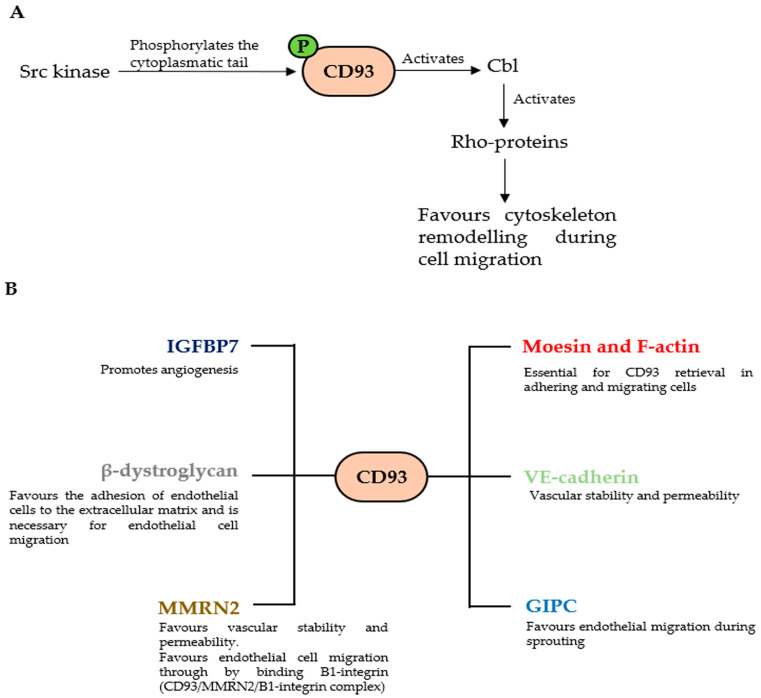
Proteins involved in CD93-mediated cytoskeleton remodeling (**A**) and proteins interacting with CD93 (**B**).

CD93 plays a key role in endothelial cell spreading since its cytoplasmic tail is phosphorylated by the Src kinase favoring the binding of the adaptor protein Cbl which recruits Crk-activating Rho-proteins such as Rac1 and RhoA that favor cytoskeleton remodeling and cell migration (Figure 3A). Moreover, CD93 exerts a key role in modulating angiogenesis, vascular stability and permeability, interacting with important proteins such as IGFBP7, MMRN2, GIMP, VE-cadherin, Moesin, F-actin and β-dystroglycan (Figure 3B).

Furthermore, the ectodomains of CD93 play a key role in cell migration since the CTLD domain of CD93 can bind MMRN2, an extracellular matrix protein [18,125], favoring cell migration. In addition, sCD93 is a potent angiogenic factor since it can induce endothelial cell differentiation, activating important pathways responsible for the cell migration, proliferation and permeability of endothelial cells such as FAK, PI3K/Akt/eNOS and ERK1/2 signaling pathways [40].

Regarding the role of CD93 in the pathologies discussed in this review, we can suggest some prospective uses of this protein as a therapeutic target. In fact, since CD93 promotes the neovascularization of choriocapillaris in patients with AMD, the use of anti-CD93 drugs (e.g., monoclonal antibodies, non-coding RNA, rottlerin and Go6976), alone or in combination with anti-VEGF therapy, may significantly improve AMD treatment and outcome. Moreover, anti-CD93 antibodies may be useful in cancer treatment since they can improve immunotherapy by increasing vascular permeability and immune cell infiltration in tumor tissue. Targeting sCD93 may also be useful in reducing inflammation in inflammatory diseases, but these drugs must be used carefully, as they may give the opposite effect. In fact, they could alter the expression of membrane-bound CD93, increasing vascular permeability and immune cell infiltration.

The evaluation of CD93 SNPs’ presence and/or sCD93 levels in patients at risk of CVD may significantly improve or avoid CVD onset through an early treatment of these patients.

In conclusion, CD93 is a key player in several normal and pathological conditions modulating important cell processes such angiogenesis, efferocytosis and inflammation. Thus, CD93 may be a potential therapeutic target in many of the diseases discussed in this review.

## Figures and Tables

**Table 1:** CD93 in angiogenesis.

Model Used	Results	Reference
Endothelial cells	CD93/β-dystroglycan interaction promoted cell migration and organization into capillary-like structures. Silencing of CD93 increased dystroglycan expression. Phosphorylation of CD93 after cell adhesion on laminin through dystroglycan was necessary for a proper endothelial migratory phenotype	[17]
Endothelial cells	rCD93D23 was more efficient in stimulating cell proliferation and migration. rCD93D23 increased MMP2 and induced cell differentiation, activating FAK, PI3K/Akt/eNOS and ERK1/2 signaling pathways promoting vessel formation	[20]
Endothelial cells	CD93 phosphorylation was Src-dependent. Phosphorylated CD93 recruited Cbl, which induced the recruitment of Crk-activating RhoA and Rac1 at the cell edge of spreading cells favoring cell motility	[31]
Endothelial cells	At onset of endothelial cell spreading, CD93 was localized to the apical bud and the cytoplasmic domain of CD93 interacted with moesin and F-actin. CD93 formed a complex with MMRN2 and active β1 integrin. The complex CD93-MMRN2- β1 integrin was moved to the basolaterally polarized cell surface through the formation of an Rab5c endosomal compartment	[34]
Endothelial cells and CD93^−/−^ mice	CD93 interacted with VE-cadherin, limiting its phosphorylation and turnover. CD93 deficiency in endothelial cells and CD93^−/−^ mice induced VE-cadherin phosphorylation, allowing its internalization and increasing barrier permeability. These effects of CD93 were due to the activation of Rho/Rho kinases	[38]
Endothelial cells and CD93^−/−^ mice	Recombinant CD93 protein promoted tube formation and sprouting of endothelial cells and accelerated wound healing in mouse subcutaneous tissue, promoting the formation of vascular-like structures. CD93 deficiency (CD93^−/−^ mice) delayed wound repair due to a reduced neovascularization of the wound area	[39]
Whole blood and PBMC from healthy subjects	MRG-110 decreased miR-92a-3p levels in whole blood and PBMC-derived CD31^+^ cells of healthy subjects. MRG-110 increased CD93 expression in whole blood	[51]
Endothelial cells	Transfection cells with MRG-110 increased CD93 expression	[52]

**Table 2:** CD93 SNPs.

Polymorphism	Alleles	Position
rs2749817	T/C/G	downstream of the 3′ UTR of CD93 genes
rs2749812	A/G	3′ UTR of CD93 genes
rs3746731	G/A/T	Exon 1 of CD93 genes

**Table 3:** CD93 in tumors.

Model Used	Results	Reference
Human glioma tissues	CD93 protein and gene expression was increased in tumor-associated blood vessels of human glioma compared with that of normal brains and was correlated to the tumor grade	[11]
Human glioblastoma tissues, GL261 glioma cell line, CD93^−/−^ mice and endothelial cells	Vascular expression of CD93 was correlated with poor survival in patients with high-grade astrocytic glioma. Tumor growth of GL261 was reduced in CD93^−/−^ mice, leading to an increased vascular permeability and decreased vascular perfusion Silencing CD93 in endothelial cells induced displacement and loss of VE-cadherin, decreasing cell–matrix adhesion, cell spreading and tube formation	[12]
NPC tissues, endothelial cells and NPC cell line CNE2	CD93 expression was increased in NPC tissues and was correlated with the T classification, N classification, distant metastasis, clinical stage and poor prognosis. Silencing CD93 in endothelial cells inhibited angiogenesis, while in the NPC cell line CNE2 reduced cell proliferation	[13]
Cancer databases	CD93 increased in tumor tissues compared with normal tissues and was associated with a poor prognosis and high TNM stage. CD93 expression was correlated with immune cell infiltration and tumor angiogenesis. Patients with low CD93 expression were more sensitive to immunotherapy	[76]
KPC, B16 cell line, endothelial cells and CD93^−/−^ mice	CD93 and IGFBP7 expression was increased in tumor-associated endothelial cells. Blockade of the CD93/IGFBP7 interaction promoted vascular maturation, reducing tumor hypoxia and increasing drug delivery	[79]
Cancer databases	An increased CD93 gene expression was associated with a poor prognosis in cancer patients. CD93 levels were correlated with MMR, TMB, MSI, immune cell infiltration, cancer onset, inflammation and angiogenesis	[80]
Human glioblastoma tissues, GL261 glioma cells and endothelial cells	CD93 was upregulated in tumor vessels of high-grade glioma. CD93 regulated β1 integrin signaling and organization of fibronectin fibrillogenesis during tumor vascularization. CD93 was expressed in endothelial filopodia and promoted their formation through the interaction with MMRN2. The CD93–MMRN2 complex was required for endothelial cell migration	[86]

NPC: nasopharyngeal carcinoma; KPC cell: mouse pancreatic adenocarcinoma; B16 cell: mouse melanoma.

**Table 4:** CD93 in inflammatory diseases.

Model Used	Results	Reference
Patients with systemic sclerosis (SSc)	Increased serum sCD93 levels in patients with SSc correlated with disease severity. sCD93 levels decreased with improvement in skin sclerosis. CD93 expression was increased on endothelial cells in lesioned skin tissues.	[23]
THP-1 cells and human primary monocytes	The mucin domain of CD93 is not necessary for its function. Recombinant human sCD93 (rhsCD93) induced cytokine production in these cells and could bind the cell surface of these latter ones. rhsCD93 induced differentiation of monocytes to macrophage-like cells and increased IL-6, IL-1β and TNF-α release. sCD93 levels in synovial fluid were high in patients with rheumatoid arthritis.	[104]
Monocytes and neutrophils isolated from healthy donors	PDBu induced the shedding of the CD93 ectodomain on monocytes, which was followed by a decreased expression of CD93 on the cell surface, while neutrophils displayed an increase in CD93 surface expression due to their replacement from intracellular stores. Inhibition of metalloproteinases blocked CD93 shedding. TNF-α and LPS stimulated CD93 shedding from monocytes.	[105]
CD93^−/−^ mice	CD93^−/−^ mice showed an increase in leukocyte infiltration during peritonitis due to an impaired vascular structure. Leukocyte recruitment and C1q hemolytic activity was restored when CD93 was expressed on either hematopoietic cells or nonhematopoietic cells in bone marrow chimeric mice. Increased levels of sCD93 in inflammatory fluid were observed only when CD93 was expressed on nonhematopoietic cells.	[107]
EAE and ADEAE neuroinflammatory mice models of neuroinflammation	CD93 was expressed in neurons, endothelial cells and microglia, while astrocytes and oligodendrocytes did not express CD93. CD93 knockout in EAE and ADEAE mice led to a more intense brain and spinal cord inflammation, and increased damage to and leakage through the blood–brain barrier compared with wild-type EAE mice.	[108]
Rat model of neuroinflammation	After inflammation induction with LPS, CD93 expression increased and then reduced, showing cytoplasm and nuclear staining in the cerebral cortex. CD93 and GIPC colocalized, but no changes in GIPC expression were found during inflammation. CD93 was expressed in microglial and neuronal cell membranes, while GIPC was expressed in both the cell membrane and cytoplasm.	[110]
U937, KHYG-1 and endothelial cells	Rottlerin downregulated CD93 expression on U937 cells. CD93 expression was downregulated by Go6976, but not by Rottlerin, in the KHYG-1 cells and by both Rottlerin and Go6976 in endothelial cells. The PKC stimulator PMA increased CD93 expression on the cell surface of all three cell lines and induced IL-8 production by the U937 cells and IFNγ production by the KHYG-1 cells. Both Go6976 and Rottlerin inhibited the upregulation of CD93 expression induced by PMA and IL-8 or IFNγ production. rTNF-α upregulated CD93 expression on the U937 cells, while rIL-1β, rIL-2, rIFNγ and LPS had no effect.	[115]

THP-1: human monocyte cell line; EAE: MOG-experimental autoimmune encephalomyelitis; ADEAE: antibody-dependent EAE; U937: human monocyte-like cell line; KHYG-1: human NK-like cell line.

## Data Availability

Not applicable.

## References

[1] McGreal E.P., Ikewaki N., Akatsu H., Morgan B.P., Gasque P. (2002). Human C1qRp is identical with CD93 and the mNI-11 antigen but does not bind C1q. J. Immunol..

[2] Khan K.A., McMurray J.L., Mohammed F., Bicknell R. (2019). C-type lectin domain group 14 proteins in vascular biology, cancer and inflammation. FEBS J..

[3] Borah S., Vasudevan D., Swain R.K. (2019). C-type lectin family XIV members and angiogenesis. Oncol. Lett..

[4] Park M., Tenner A.J. (2003). Cell surface expression of C1qRP/CD93 is stabilized by O-glycosylation. J. Cell. Physiol..

[5] Nepomuceno R.R., Tenner A.J. (1998). C1qRP, the C1q receptor that enhances phagocytosis, is detected specifically in human cells of myeloid lineage, endothelial cells, and platelets. J. Immunol..

[6] Lovik G., Larsen Sand K., Iversen J.G., Rolstad B. (2001). C1qRp elicits a Ca^++^ response in rat NK cells but does not influence NK-mediated cytotoxicity. Scand. J. Immunol..

[7] Danet G.H., Luongo J.L., Butler G., Lu M.M., Tenner A.J., Simon M.C., Bonnet D.A. (2002). C1qRp defines a new human stem cell population with hematopoietic and hepatic potential. Proc. Natl. Acad. Sci. USA.

[8] Ikewaki N., Yamao H., Kulski J.K., Inoko H. (2010). Flow cytometric identification of CD93 expression on naive T lymphocytes (CD4^+^CD45RA^+^ cells) in human neonatal umbilical cord blood. J. Clin. Immunol..

[9] Chevrier S., Genton C., Kallies A., Karnowski A., Otten L.A., Malissen B., Malissen M., Botto M., Corcoran L.M., Nutt S.L. (2009). CD93 is required for maintenance of antibody secretion and persistence of plasma cells in the bone marrow niche. Proc. Natl. Acad. Sci. USA.

[10] Olsen R.S., Lindh M., Vorkapic E., Andersson R.E., Zar N., Lofgren S., Dimberg J., Matussek A., Wagsater D. (2015). CD93 gene polymorphism is associated with disseminated colorectal cancer. Int. J. Color. Dis..

[11] Dieterich L.C., Mellberg S., Langenkamp E., Zhang L., Zieba A., Salomaki H., Teichert M., Huang H., Edqvist P.H., Kraus T. (2012). Transcriptional profiling of human glioblastoma vessels indicates a key role of VEGF-A and TGFbeta2 in vascular abnormalization. J. Pathol..

[12] Langenkamp E., Zhang L., Lugano R., Huang H., Elhassan T.E., Georganaki M., Bazzar W., Loof J., Trendelenburg G., Essand M. (2015). Elevated expression of the C-type lectin CD93 in the glioblastoma vasculature regulates cytoskeletal rearrangements that enhance vessel function and reduce host survival. Cancer Res..

[13] Bao L., Tang M., Zhang Q., You B., Shan Y., Shi S., Li L., Hu S., You Y. (2016). Elevated expression of CD93 promotes angiogenesis and tumor growth in nasopharyngeal carcinoma. Biochem. Biophys. Res. Commun..

[14] Lugano R., Ramachandran M., Dimberg A. (2020). Tumor angiogenesis: Causes, consequences, challenges and opportunities. Cell. Mol. Life Sci..

[15] Orlandini M., Galvagni F., Bardelli M., Rocchigiani M., Lentucci C., Anselmi F., Zippo A., Bini L., Oliviero S. (2014). The characterization of a novel monoclonal antibody against CD93 unveils a new antiangiogenic target. Oncotarget.

[16] Ayers M., Fargnoli J., Lewin A., Wu Q., Platero J.S. (2007). Discovery and validation of biomarkers that respond to treatment with brivanib alaninate, a small-molecule VEGFR-2/FGFR-1 antagonist. Cancer Res..

[17] Galvagni F., Nardi F., Maida M., Bernardini G., Vannuccini S., Petraglia F., Santucci A., Orlandini M. (2016). CD93 and dystroglycan cooperation in human endothelial cell adhesion and migration adhesion and migration. Oncotarget.

[18] Khan K.A., Naylor A.J., Khan A., Noy P.J., Mambretti M., Lodhia P., Athwal J., Korzystka A., Buckley C.D., Willcox B.E. (2017). Multimerin-2 is a ligand for group 14 family C-type lectins CLEC14A, CD93 and CD248 spanning the endothelial pericyte interface. Oncogene.

[19] Galvagni F., Nardi F., Spiga O., Trezza A., Tarticchio G., Pellicani R., Andreuzzi E., Caldi E., Toti P., Tosi G.M. (2017). Dissecting the CD93-Multimerin 2 interaction involved in cell adhesion and migration of the activated endothelium. Matrix Biol..

[20] Kao Y.C., Jiang S.J., Pan W.A., Wang K.C., Chen P.K., Wei H.J., Chen W.S., Chang B.I., Shi G.Y., Wu H.L. (2012). The epidermal growth factor-like domain of CD93 is a potent angiogenic factor. PLoS ONE.

[21] Linton M.F., Babaev V.R., Huang J., Linton E.F., Tao H., Yancey P.G. (2016). Macrophage Apoptosis and Efferocytosis in the Pathogenesis of Atherosclerosis. Circ. J..

[22] Lee M., Park H.S., Choi M.Y., Kim H.Z., Moon S.J., Ha J.Y., Choi A., Park Y.W., Park J.S., Shin E.C. (2020). Significance of Soluble CD93 in Type 2 Diabetes as a Biomarker for Diabetic Nephropathy: Integrated Results from Human and Rodent Studies. J. Clin. Med..

[23] Yanaba K., Asano Y., Noda S., Akamata K., Aozasa N., Taniguchi T., Takahashi T., Ichimura Y., Toyama T., Sumida H. (2012). Augmented production of soluble CD93 in patients with systemic sclerosis and clinical association with severity of skin sclerosis. Br. J. Dermatol..

[24] van der Net J.B., Oosterveer D.M., Versmissen J., Defesche J.C., Yazdanpanah M., Aouizerat B.E., Steyerberg E.W., Malloy M.J., Pullinger C.R., Kastelein J.J. (2008). Replication study of 10 genetic polymorphisms associated with coronary heart disease in a specific high-risk population with familial hypercholesterolemia. Eur. Heart J..

[25] Norsworthy P.J., Fossati-Jimack L., Cortes-Hernandez J., Taylor P.R., Bygrave A.E., Thompson R.D., Nourshargh S., Walport M.J., Botto M. (2004). Murine CD93 (C1qRp) contributes to the removal of apoptotic cells in vivo but is not required for C1q-mediated enhancement of phagocytosis. J. Immunol..

[26] Lamalice L., Le Boeuf F., Huot J. (2007). Endothelial cell migration during angiogenesis. Circ. Res..

[27] Szczesny-Malysiak E., Stojak M., Campagna R., Grosicki M., Jamrozik M., Kaczara P., Chlopicki S. (2020). Bardoxolone Methyl Displays Detrimental Effects on Endothelial Bioenergetics, Suppresses Endothelial ET-1 Release, and Increases Endothelial Permeability in Human Microvascular Endothelium. Oxid. Med. Cell. Longev..

[28] Campagna R., Mateuszuk L., Wojnar-Lason K., Kaczara P., Tworzydlo A., Kij A., Bujok R., Mlynarski J., Wang Y., Sartini D. (2021). Nicotinamide *N*-methyltransferase in endothelium protects against oxidant stress-induced endothelial injury. Biochim. Biophys. Acta Mol. Cell Res..

[29] Barbera S., Lugano R., Pedalina A., Mongiat M., Santucci A., Tosi G.M., Dimberg A., Galvagni F., Orlandini M. (2021). The C-type lectin CD93 controls endothelial cell migration via activation of the Rho family of small GTPases. Matrix Biol..

[30] Nakashima N., Rose D.W., Xiao S., Egawa K., Martin S.S., Haruta T., Saltiel A.R., Olefsky J.M. (1999). The functional role of CrkII in actin cytoskeleton organization and mitogenesis. J. Biol. Chem..

[31] Barbera S., Raucci L., Lugano R., Tosi G.M., Dimberg A., Santucci A., Galvagni F., Orlandini M. (2021). CD93 Signaling via Rho Proteins Drives Cytoskeletal Remodeling in Spreading Endothelial Cells. Int. J. Mol. Sci..

[32] Ebnet K., Kummer D., Steinbacher T., Singh A., Nakayama M., Matis M. (2018). Regulation of cell polarity by cell adhesion receptors. Semin. Cell. Dev. Biol..

[33] Galvagni F., Baldari C.T., Oliviero S., Orlandini M. (2012). An apical actin-rich domain drives the establishment of cell polarity during cell adhesion. Histochem. Cell Biol..

[34] Barbera S., Nardi F., Elia I., Realini G., Lugano R., Santucci A., Tosi G.M., Dimberg A., Galvagni F., Orlandini M. (2019). The small GTPase Rab5c is a key regulator of trafficking of the CD93/Multimerin-2/beta1 integrin complex in endothelial cell adhesion and migration. Cell Commun. Signal..

[35] De Franceschi N., Hamidi H., Alanko J., Sahgal P., Ivaska J. (2015). Integrin traffic–the update. J. Cell Sci..

[36] Sandri C., Caccavari F., Valdembri D., Camillo C., Veltel S., Santambrogio M., Lanzetti L., Bussolino F., Ivaska J., Serini G. (2012). The R-Ras/RIN2/Rab5 complex controls endothelial cell adhesion and morphogenesis via active integrin endocytosis and Rac signaling. Cell Res..

[37] Claesson-Welsh L., Dejana E., McDonald D.M. (2021). Permeability of the Endothelial Barrier: Identifying and Reconciling Controversies. Trends Mol. Med..

[38] Lugano R., Vemuri K., Barbera S., Orlandini M., Dejana E., Claesson-Welsh L., Dimberg A. (2023). CD93 maintains endothelial barrier function by limiting the phosphorylation and turnover of VE-cadherin. FASEB J..

[39] Xu Y., Jia Y., Wu N., Wang J., He L., Yang D. CD93 Ameliorates Diabetic Wounds by Promoting Angiogenesis via the p38MAPK/MK2/HSP27 Axis. Eur. J. Vasc. Endovasc. Surg..

[40] Chung B.H., Cho Y.L., Kim J.D., Jo H.S., Won M.H., Lee H., Ha K.S., Kwon Y.G., Kim Y.M. (2010). Promotion of direct angiogenesis in vitro and in vivo by Puerariae flos extract via activation of MEK/ERK-, PI3K/Akt/eNOS-, and Src/FAK-dependent pathways. Phytother. Res..

[41] Avellini C., Licini C., Lazzarini R., Gesuita R., Guerra E., Tossetta G., Castellucci C., Giannubilo S.R., Procopio A., Alberti S. (2017). The trophoblast cell surface antigen 2 and miR-125b axis in urothelial bladder cancer. Oncotarget.

[42] Karimi Dermani F., Datta I., Gholamzadeh Khoei S. (2023). MicroRNA-452: A double-edged sword in multiple human cancers. Clin. Transl. Oncol..

[43] Li L., Xun C., Yu C.H. (2022). Role of microRNA-regulated cancer stem cells in recurrent hepatocellular carcinoma. World J. Hepatol..

[44] Poniewierska-Baran A., Sluczanowska-Glabowska S., Malkowska P., Sierawska O., Zadroga L., Pawlik A., Niedzwiedzka-Rystwej P. (2022). Role of miRNA in Melanoma Development and Progression. Int. J. Mol. Sci..

[45] Licini C., Avellini C., Picchiassi E., Mensa E., Fantone S., Ramini D., Tersigni C., Tossetta G., Castellucci C., Tarquini F. (2021). Pre-eclampsia predictive ability of maternal miR-125b: A clinical and experimental study. Transl. Res..

[46] Atic A.I., Thiele M., Munk A., Dalgaard L.T. (2023). Circulating microRNAs associated with non-alcoholic fatty liver disease. Am. J. Physiol. Cell Physiol..

[47] Elkhawaga S.Y., Ismail A., Elsakka E.G.E., Doghish A.S., Elkady M.A., El-Mahdy H.A. (2023). miRNAs as cornerstones in adipogenesis and obesity. Life Sci..

[48] Tofigh R., Hosseinpourfeizi M., Baradaran B., Teimourian S., Safaralizadeh R. (2023). Rheumatoid arthritis and non-coding RNAs; how to trigger inflammation. Life Sci..

[49] Kumar S., Kim C.W., Simmons R.D., Jo H. (2014). Role of flow-sensitive microRNAs in endothelial dysfunction and atherosclerosis: Mechanosensitive athero-miRs. Arterioscler. Thromb. Vasc. Biol..

[50] Anand S., Cheresh D.A. (2011). MicroRNA-mediated regulation of the angiogenic switch. Curr. Opin. Hematol..

[51] Abplanalp W.T., Fischer A., John D., Zeiher A.M., Gosgnach W., Darville H., Montgomery R., Pestano L., Allee G., Paty I. (2020). Efficiency and Target Derepression of Anti-miR-92a: Results of a First in Human Study. Nucleic Acid. Ther..

[52] Gallant-Behm C.L., Piper J., Dickinson B.A., Dalby C.M., Pestano L.A., Jackson A.L. (2018). A synthetic microRNA-92a inhibitor (MRG-110) accelerates angiogenesis and wound healing in diabetic and nondiabetic wounds. Wound Repair. Regen..

[53] Collins F.S., Guyer M.S., Charkravarti A. (1997). Variations on a theme: Cataloging human DNA sequence variation. Science.

[54] Gray N.K. (1998). Translational control by repressor proteins binding to the 5′UTR of mRNAs. Methods Mol. Biol..

[55] Tossetta G., Fantone S., Montanari E., Marzioni D., Goteri G. (2022). Role of NRF2 in Ovarian Cancer. Antioxidants.

[56] Malarstig A., Silveira A., Wagsater D., Ohrvik J., Backlund A., Samnegard A., Khademi M., Hellenius M.L., Leander K., Olsson T. (2011). Plasma CD93 concentration is a potential novel biomarker for coronary artery disease. J. Intern. Med..

[57] Gaspari A.A. (2006). Innate and adaptive immunity and the pathophysiology of psoriasis. J. Am. Acad. Dermatol..

[58] Lowes M.A., Bowcock A.M., Krueger J.G. (2007). Pathogenesis and therapy of psoriasis. Nature.

[59] Shehata W.A., Maraee A.H., Tayel N., Mohamed A.S., Abd El Gayed E.M., Elsayed N., Mostafa M.I., Bazid H.A.S. (2022). CD93 has a crucial role in pathogenesis of psoriasis. J. Cosmet. Dermatol..

[60] Duvetorp A., Slind Olsen R., Skarstedt M., Soderman J., Seifert O. (2017). Psoriasis and Pro-angiogenetic Factor CD93: Gene Expression and Association with Gene Polymorphism Suggests a Role in Disease Pathogenesis. Acta Derm. Venereol..

[61] Alehagen U., Shamoun L., Wagsater D. (2020). Genetic variance and plasma concentration of CD93 is associated with cardiovascular mortality: Results from a 6.7-year follow-up of a healthy community-living elderly population. Mol. Med. Rep..

[62] Flaxman S.R., Bourne R.R.A., Resnikoff S., Ackland P., Braithwaite T., Cicinelli M.V., Das A., Jonas J.B., Keeffe J., Kempen J.H. (2017). Global causes of blindness and distance vision impairment 1990-2020: A systematic review and meta-analysis. Lancet Glob. Health.

[63] Wong W.L., Su X., Li X., Cheung C.M., Klein R., Cheng C.Y., Wong T.Y. (2014). Global prevalence of age-related macular degeneration and disease burden projection for 2020 and 2040: A systematic review and meta-analysis. Lancet Glob. Health.

[64] Coleman H.R., Chan C.C., Ferris F.L., Chew E.Y. (2008). Age-related macular degeneration. Lancet.

[65] Handa J.T., Bowes Rickman C., Dick A.D., Gorin M.B., Miller J.W., Toth C.A., Ueffing M., Zarbin M., Farrer L.A. (2019). A systems biology approach towards understanding and treating non-neovascular age-related macular degeneration. Nat. Commun..

[66] Servillo A., Zucchiatti I., Sacconi R., Parravano M., Querques L., La Rubia P., Prascina F., Bandello F., Querques G. (2023). The state-of-the-art pharmacotherapeutic management of neovascular age-related macular degeneration. Expert. Opin. Pharmacother..

[67] Tosi G.M., Neri G., Barbera S., Mundo L., Parolini B., Lazzi S., Lugano R., Poletto E., Leoncini L., Pertile G. (2020). The Binding of CD93 to Multimerin-2 Promotes Choroidal Neovascularization. Investig. Ophthalmol. Vis. Sci..

[68] Tosi G.M., Caldi E., Parolini B., Toti P., Neri G., Nardi F., Traversi C., Cevenini G., Marigliani D., Nuti E. (2017). CD93 as a Potential Target in Neovascular Age-Related Macular Degeneration. J. Cell. Physiol..

[69] Guillaume Z., Auvray M., Vano Y., Oudard S., Helley D., Mauge L. (2022). Renal Carcinoma and Angiogenesis: Therapeutic Target and Biomarkers of Response in Current Therapies. Cancers.

[70] Zheng W., Qian C., Tang Y., Yang C., Zhou Y., Shen P., Chen W., Yu S., Wei Z., Wang A. (2022). Manipulation of the crosstalk between tumor angiogenesis and immunosuppression in the tumor microenvironment: Insight into the combination therapy of anti-angiogenesis and immune checkpoint blockade. Front. Immunol..

[71] Pozzi V., Campagna R., Sartini D., Emanuelli M. (2022). Nicotinamide *N*-Methyltransferase as Promising Tool for Management of Gastrointestinal Neoplasms. Biomolecules.

[72] Guo S., Feng J., Li Z., Yang S., Qiu X., Xu Y., Shen Z. (2023). Improved cancer immunotherapy strategies by nanomedicine. Wiley Interdiscip. Rev. Nanomed. Nanobiotechnol..

[73] Sharma P., Hu-Lieskovan S., Wargo J.A., Ribas A. (2017). Primary, Adaptive, and Acquired Resistance to Cancer Immunotherapy. Cell.

[74] Guo A., Zhang J., Tian Y., Peng Y., Luo P., Zhang J., Liu Z., Wu W., Zhang H., Cheng Q. (2022). Identify the immune characteristics and immunotherapy value of CD93 in the pan-cancer based on the public data sets. Front. Immunol..

[75] Campagna R., Pozzi V., Spinelli G., Sartini D., Milanese G., Galosi A.B., Emanuelli M. (2021). The Utility of Nicotinamide *N*-Methyltransferase as a Potential Biomarker to Predict the Oncological Outcomes for Urological Cancers: An Update. Biomolecules.

[76] Tong W., Wang G., Zhu L., Bai Y., Liu Z., Yang L., Wu H., Cui T., Zhang Y. (2021). Pan-Cancer Analysis Identified CD93 as a Valuable Biomarker for Predicting Patient Prognosis and Immunotherapy Response. Front. Mol. Biosci..

[77] Passaro A., Stenzinger A., Peters S. (2020). Tumor Mutational Burden as a Pan-cancer Biomarker for Immunotherapy: The Limits and Potential for Convergence. Cancer Cell.

[78] Samstein R.M., Lee C.H., Shoushtari A.N., Hellmann M.D., Shen R., Janjigian Y.Y., Barron D.A., Zehir A., Jordan E.J., Omuro A. (2019). Tumor mutational load predicts survival after immunotherapy across multiple cancer types. Nat. Genet..

[79] Sun Y., Chen W., Torphy R.J., Yao S., Zhu G., Lin R., Lugano R., Miller E.N., Fujiwara Y., Bian L. (2021). Blockade of the CD93 pathway normalizes tumor vasculature to facilitate drug delivery and immunotherapy. Sci. Transl. Med..

[80] Zhang Z., Zheng M., Ding Q., Liu M. (2022). CD93 Correlates with Immune Infiltration and Impacts Patient Immunotherapy Efficacy: A Pan-Cancer Analysis. Front. Cell Dev. Biol..

[81] Dreher M.R., Liu W., Michelich C.R., Dewhirst M.W., Yuan F., Chilkoti A. (2006). Tumor vascular permeability, accumulation, and penetration of macromolecular drug carriers. J. Natl. Cancer Inst..

[82] Lanitis E., Irving M., Coukos G. (2015). Targeting the tumor vasculature to enhance T cell activity. Curr. Opin. Immunol..

[83] Hatfield S.M., Kjaergaard J., Lukashev D., Schreiber T.H., Belikoff B., Abbott R., Sethumadhavan S., Philbrook P., Ko K., Cannici R. (2015). Immunological mechanisms of the antitumor effects of supplemental oxygenation. Sci. Transl. Med..

[84] Emanuelli M., Sartini D., Molinelli E., Campagna R., Pozzi V., Salvolini E., Simonetti O., Campanati A., Offidani A. (2022). The Double-Edged Sword of Oxidative Stress in Skin Damage and Melanoma: From Physiopathology to Therapeutical Approaches. Antioxidants.

[85] Jain R.K., di Tomaso E., Duda D.G., Loeffler J.S., Sorensen A.G., Batchelor T.T. (2007). Angiogenesis in brain tumours. Nat. Rev. Neurosci..

[86] Lugano R., Vemuri K., Yu D., Bergqvist M., Smits A., Essand M., Johansson S., Dejana E., Dimberg A. (2018). CD93 promotes beta1 integrin activation and fibronectin fibrillogenesis during tumor angiogenesis. J. Clin. Investig..

[87] Nathan C. (2002). Points of control in inflammation. Nature.

[88] Marinelli Busilacchi E., Costantini A., Mancini G., Tossetta G., Olivieri J., Poloni A., Viola N., Butini L., Campanati A., Goteri G. (2020). Nilotinib Treatment of Patients Affected by Chronic Graft-versus-Host Disease Reduces Collagen Production and Skin Fibrosis by Downmodulating the TGF-beta and p-SMAD Pathway. Biol. Blood Marrow Transplant..

[89] Tossetta G., Fantone S., Gesuita R., Di Renzo G.C., Meyyazhagan A., Tersigni C., Scambia G., Di Simone N., Marzioni D. (2022). HtrA1 in Gestational Diabetes Mellitus: A Possible Biomarker?. Diagnostics.

[90] Rai U., Senapati D., Arora M.K. (2023). Insights on the role of anti-inflammatory and immunosuppressive agents in the amelioration of diabetes. Diabetol. Int..

[91] Tossetta G., Fantone S., Licini C., Marzioni D., Mattioli-Belmonte M. (2022). The multifaced role of HtrA1 in the development of joint and skeletal disorders. Bone.

[92] Johnson N.H., de Rivero Vaccari J.P., Bramlett H.M., Keane R.W., Dietrich W.D. (2023). Inflammasome activation in traumatic brain injury and Alzheimer’s disease. Transl. Res..

[93] Charabati M., Wheeler M.A., Weiner H.L., Quintana F.J. (2023). Multiple sclerosis: Neuroimmune crosstalk and therapeutic targeting. Cell.

[94] Wadstrom B.N., Pedersen K.M., Wulff A.B., Nordestgaard B.G. (2023). Inflammation compared to low-density lipoprotein cholesterol: Two different causes of atherosclerotic cardiovascular disease. Curr. Opin. Lipidol..

[95] Monsour M., Borlongan C.V. (2023). The central role of peripheral inflammation in ischemic stroke. J. Cereb. Blood Flow. Metab..

[96] Fantone S., Giannubilo S.R., Marzioni D., Tossetta G. (2021). HTRA family proteins in pregnancy outcome. Tissue Cell.

[97] Licini C., Tossetta G., Avellini C., Ciarmela P., Lorenzi T., Toti P., Gesuita R., Voltolini C., Petraglia F., Castellucci M. (2016). Analysis of cell-cell junctions in human amnion and chorionic plate affected by chorioamnionitis. Histol. Histopathol..

[98] Tossetta G., Fantone S., Delli Muti N., Balercia G., Ciavattini A., Giannubilo S.R., Marzioni D. (2022). Preeclampsia and severe acute respiratory syndrome coronavirus 2 infection: A systematic review. J. Hypertens..

[99] Tossetta G., Fantone S., Giannubilo S.R., Marzioni D. (2021). The Multifaced Actions of Curcumin in Pregnancy Outcome. Antioxidants.

[100] Tossetta G. (2022). Metformin Improves Ovarian Cancer Sensitivity to Paclitaxel and Platinum-Based Drugs: A Review of In Vitro Findings. Int. J. Mol. Sci..

[101] Peczek P., Gajda M., Rutkowski K., Fudalej M., Deptala A., Badowska-Kozakiewicz A.M. (2023). Cancer-associated inflammation: Pathophysiology and clinical significance. J. Cancer Res. Clin. Oncol..

[102] Altobelli E., Latella G., Morroni M., Licini C., Tossetta G., Mazzucchelli R., Profeta V.F., Coletti G., Leocata P., Castellucci M. (2017). Low HtrA1 expression in patients with long-standing ulcerative colitis and colorectal cancer. Oncol. Rep..

[103] Haubruck P., Pinto M.M., Moradi B., Little C.B., Gentek R. (2021). Monocytes, Macrophages, and Their Potential Niches in Synovial Joints—Therapeutic Targets in Post-Traumatic Osteoarthritis?. Front. Immunol..

[104] Jeon J.W., Jung J.G., Shin E.C., Choi H.I., Kim H.Y., Cho M.L., Kim S.W., Jang Y.S., Sohn M.H., Moon J.H. (2010). Soluble CD93 induces differentiation of monocytes and enhances TLR responses. J. Immunol..

[105] Bohlson S.S., Silva R., Fonseca M.I., Tenner A.J. (2005). CD93 is rapidly shed from the surface of human myeloid cells and the soluble form is detected in human plasma. J. Immunol..

[106] Ojo A.B., Omoareghan Irabor D. (2022). Bacterial and Antibiotic Sensitivity Pattern in Secondary Peritonitis. J. West Afr. Coll. Surg..

[107] Greenlee-Wacker M.C., Briseno C., Galvan M., Moriel G., Velazquez P., Bohlson S.S. (2011). Membrane-associated CD93 regulates leukocyte migration and C1q-hemolytic activity during murine peritonitis. J. Immunol..

[108] Griffiths M.R., Botto M., Morgan B.P., Neal J.W., Gasque P. (2018). CD93 regulates central nervous system inflammation in two mouse models of autoimmune encephalomyelitis. Immunology.

[109] Bohlson S.S., Zhang M., Ortiz C.E., Tenner A.J. (2005). CD93 interacts with the PDZ domain-containing adaptor protein GIPC: Implications in the modulation of phagocytosis. J. Leukoc. Biol..

[110] Liu C., Cui Z., Wang S., Zhang D. (2014). CD93 and GIPC expression and localization during central nervous system inflammation. Neural Regen. Res..

[111] Volkmann E.R., Andreasson K., Smith V. (2023). Systemic sclerosis. Lancet.

[112] Poole A.W., Pula G., Hers I., Crosby D., Jones M.L. (2004). PKC-interacting proteins: From function to pharmacology. Trends Pharmacol. Sci..

[113] Idris I., Gray S., Donnelly R. (2001). Protein kinase C activation: Isozyme-specific effects on metabolism and cardiovascular complications in diabetes. Diabetologia.

[114] Spitaler M., Cantrell D.A. (2004). Protein kinase C and beyond. Nat. Immunol..

[115] Ikewaki N., Kulski J.K., Inoko H. (2006). Regulation of CD93 cell surface expression by protein kinase C isoenzymes. Microbiol. Immunol..

[116] Benincasa G., Coscioni E., Napoli C. (2022). Cardiovascular risk factors and molecular routes underlying endothelial dysfunction: Novel opportunities for primary prevention. Biochem. Pharmacol..

[117] Piani F., Tossetta G., Cara-Fuentes G., Agnoletti D., Marzioni D., Borghi C. (2023). Diagnostic and Prognostic Role of CD93 in Cardiovascular Disease: A Systematic Review. Biomolecules.

[118] Sharma K., Chanana N., Mohammad G., Thinlas T., Gupta M., Syed M.A., Das R.S., Pasha Q., Mishra A. (2021). Hypertensive Patients Exhibit Enhanced Thrombospondin-1 Levels at High-Altitude. Life.

[119] Adamski M.G., Li Y., Wagner E., Yu H., Seales-Bailey C., Soper S.A., Murphy M., Baird A.E. (2014). Expression profile based gene clusters for ischemic stroke detection. Genomics.

[120] Bicvic A., Scherrer N., Schweizer J., Fluri F., Christ-Crain M., De Marchis G.M., Luft A.R., Katan M. (2022). A novel biomarker panel index improves risk stratification after ischemic stroke. Eur. Stroke J..

[121] Youn J.C., Yu H.T., Jeon J.W., Lee H.S., Jang Y., Park Y.W., Park Y.B., Shin E.C., Ha J.W. (2014). Soluble CD93 levels in patients with acute myocardial infarction and its implication on clinical outcome. PLoS ONE.

[122] Strawbridge R.J., Hilding A., Silveira A., Osterholm C., Sennblad B., McLeod O., Tsikrika P., Foroogh F., Tremoli E., Baldassarre D. (2016). Soluble CD93 Is Involved in Metabolic Dysregulation but Does Not Influence Carotid Intima-Media Thickness. Diabetes.

[123] Helleberg S., Engel A., Ahmed S., Ahmed A., Rådegran G. (2022). Higher plasma IL-6 and PTX3 are associated with worse survival in left heart failure with pulmonary hypertension. Am. Heart J. Plus Cardiol. Res. Pract..

[124] Bouwens E., van den Berg V.J., Akkerhuis K.M., Baart S.J., Caliskan K., Brugts J.J., Mouthaan H., Ramshorst J.V., Germans T., Umans V. (2020). Circulating Biomarkers of Cell Adhesion Predict Clinical Outcome in Patients with Chronic Heart Failure. J. Clin. Med..

[125] Fejza A., Poletto E., Carobolante G., Camicia L., Andreuzzi E., Capuano A., Pivetta E., Pellicani R., Colladel R., Marastoni S. (2021). Multimerin-2 orchestrates the cross-talk between endothelial cells and pericytes: A mechanism to maintain vascular stability. Matrix Biol. Plus.

